# Molecular Insights of SARS-CoV-2 Antivirals Administration: A Balance between Safety Profiles and Impact on Cardiovascular Phenotypes

**DOI:** 10.3390/biomedicines10020437

**Published:** 2022-02-14

**Authors:** Francesco Nappi, Adelaide Iervolino, Sanjeet Singh Avtaar Singh

**Affiliations:** 1 Department of Cardiac Surgery, Centre Cardiologique du Nord de Saint-Denis, 93200 Saint-Denis, France; 2 Department of Internal Medicine, University Policlinic Federico II, 80131 Naples, Italy; adelaide.iervolino@libero.it; 3 Department of Cardiothoracic Surgery, Aberdeen Royal Infirmary, Aberdeen AB25 2ZN, UK; sanjeetsingh@nhs.scot

**Keywords:** SARS-CoV-2, antivirals, arrhythmias, DDIs, pharmacokinetics, safety profile

## Abstract

The COVID-19 pandemic has resulted in a complex clinical challenge, caused by a novel coronavirus, partially similar to previously known coronaviruses but with a different pattern of contagiousness, complications, and mortality. Since its global spread, several therapeutic agents have been developed to address the heterogeneous disease treatment, in terms of severity, hospital or outpatient management, and pre-existing clinical conditions. To better understand the rationale of new or old repurposed medications, the structure and host–virus interaction molecular bases are presented. The recommended agents by EDSA guidelines comprise of corticosteroids, JAK-targeting monoclonal antibodies, IL-6 inhibitors, and antivirals, some of them showing narrow indications due to the lack of large population trials and statistical power. The aim of this review is to present FDA-approved or authorized for emergency use antivirals, namely remdesivir, molnupinavir, and the combination nirmatrelvir-ritonavir and their impact on the cardiovascular system. We reviewed the literature for metanalyses, randomized clinical trials, and case reports and found positive associations between remdesivir and ritonavir administration at therapeutic doses and changes in cardiac conduction, relatable to their previously known pro-arrhythmogenic effects and important ritonavir interactions with cardioactive medications including antiplatelets, anti-arrhythmic agents, and lipid-lowering drugs, possibly interfering with pre-existing therapeutic regimens. Nonetheless, safety profiles of antivirals are largely questioned and addressed by health agencies, in consideration of COVID-19 cardiac and pro-thrombotic complications generally experienced by predisposed subjects. Our advice is to continuously adhere to the strict indications of FDA documents, monitor the possible side effects of antivirals, and increase physicians’ awareness on the co-administration of antivirals and cardiovascular-relevant medications. This review dissects the global and local tendency to structure patient-based treatment plans, for a glance towards practical application of precision medicine.

## 1. Introduction: COVID-19 Pandemic, a Novel Biological Entity for a Modern Clinical Challenge

### 1.1. SARS-CoV-2 Global Effects and Possible Management Strategies

The COVID-19 global pandemic has involved 288,195,906 confirmed cases, including 5,454,751 deaths, as reported by World Health Organization (WHO) as of 31 December 2021 [1].

The causative infective agent is a novel coronavirus consisting of a single strand of positive-sense RNA, initially termed “severe acute respiratory syndrome coronavirus 2” (SARS-CoV-2) by the Coronaviridae Study Group (CSG) of the International Committee on Taxonomy of Viruses in February 2020 [2,3,4].

Previously, two other coronaviruses, SARS-CoV and MERS-CoV, were identified as belonging to the same genus and responsible for fast-spreading epidemics. Since then, scientists have investigated and pointed out the differences in mortality and contagiousness rates that linked these three human coronaviruses. While SARS-CoV and MERS-CoV reported death rates of 10–15% and 37% respectively, SARS-CoV-2 showed a lower mortality rate than the former two [5]. Other major differences include longer incubation time after initial infection (1–2 weeks) and a more conspicuous reservoir of infected people spreading SARS-CoV-2 in an asymptomatic state.

The World Health Organization has indicated Europe as the epicenter of the pandemic recovery from SARS-CoV-2 for the winter of 2022 [1]. The arduous task of curbing the thrust of the pandemic has directed scientists towards an acceleration in research programs, obtaining extraordinary results in a short time.

The researchers’ work led to the development, among others, of BNT162b2 mRNA and mRNA-1273 vaccines developed by Pfizer/BioNtech and Moderna companies and of Ad26.COV2.S, a recombinant, replication-incompetent human adenoviral vector by Johnson and Johnson which has been approved by the Food and Drug Administration (FDA) [6,7,8]. Concerning therapeutic agents, small molecule antiviral drugs including remdesivir and molnupinavir have been clinically tested. Remdesivir, after its approval on 22 October 2020, has already been used according to guidelines [9].

Moreover, evidence has shown that SARS-CoV-2 continues to mutate at a rate of approximately 10^6^ per site per cycle resulting in several variants with uncontrolled dissemination among humans [10,11]. There were suggestions that the vaccines, rather than therapeutic agents, proved less effective on virus mutations [12]. Furthermore, with the possible emergence of future coronavirus variants, the development of new antiviral agents remains pivotal in terms of preparing for new outbreaks.

Simultaneously, severe respiratory and cardiovascular symptoms, among certain vulnerable populations, have raised concerns with an emphasis on clinical attempts to diminish the risk of spread, especially to these cohorts.

Recently, investigators have focused their attention on cardiac and thrombotic complications, to which clot-promoting autoantibodies are likely to arise. Zuo et al. [13] drew on previous evidence by highlighting points of convergence between blood clotting anomalies in patients with COVID-19 and those with autoimmune clotting clinical condition such as antiphospholipid syndrome (APS).

It is vital to therefore consider antivirals’ safety, mostly to implement existing guidelines and counterbalance unrestrained viral replication effects, responsible for the inflammatory complications [14]. However, the role of antivirals should not be to replace vaccines but rather to act as additional complements for the treatment of infection caused by the wild-type parent virus (WT) than for that caused by variants.

Evidence suggests that some of these viral replication inhibitors met the validity criteria for consideration by major American and European drug agencies. We deeply believe structural and functional details of SARS-CoV-2 are fundamental for understanding the ongoing efforts to implement antiviral therapies.

### 1.2. Structure, Genomics, and Viral Particle–Host Interaction

SARS-CoV-2 has a spherical shape and multiple components are essential for its replication and transcription: several club-like projections on the surface of the envelope representing the spike glycoprotein (S), which permits the adhesion to the host cell and induces antibody neutralization; the envelope protein membrane (E); the structural membrane protein (M), which is across the lipid bilayer; the hemagglutinin-esterase glycoprotein (HE), which interacts with the sialic acid on the host cell inactivating it and helping the virus inject its genetic material inside cellular cytoplasm; the nucleoprotein (N); and the positive-sense single-stranded RNA [15,16,17].

The genome size of COVID-19 is about 26.4–31.7 Kb (kilobases). Inside the viral genome, several open reading frames (ORF) can be observed along with untranslated regions (UTR). ORF1a/b (29.8 Kb size) encodes the replicase polyprotein named pp1a protein and 16 accessory non-structural proteins (nsps) while ORF1b codes for pp1b and 10 nsps [18,19,20,21]. ORFs 10 and 11 encode structural proteins such as spike (S), envelope (E), membrane protein (M), and nucleoprotein (N). ORF3, ORF7a, ORF7b, and ORF8 produce other essential accessory proteins [22].

SARS-CoV-2 has some genomic differences compared to SARS-CoV and MERS-CoV. Some of the variabilities make SARS-CoV-2 more virulent than the other two viruses. A single mutation at N501T in the spike protein enhances the binding efficiency of SARS-CoV-2 to Angiotensin-Converting Enzyme 2 (ACE2) [23].

To date, we have identified six different clades of SARS-CoV-2: (1) L clade (originated in Wuhan, China and the similar ORF8-L84S clade), (2) S clade (mutation of ORF8, L84S), (3) V clade (variant of ORF3a coding protein NS3, G251V), (4) G clade (mutation in spike protein, D614G), (5) GH clade (mutations in spike protein, D614G and ORF3a, Q57H), and (6) GR clade (mutation in nucleocapsid gene, RG203KR) [24,25]. G and GR clades are predominant in Europe, while GH is more common in North America.

Once in the host system, SARS-CoV-2 enters the cell. The spike protein binds the virus to ACE2 receptor on the surface of the cell [26], it fuses to the cell membrane and enters into the cytoplasm by endosomic transport. Once inside the cell, the virus releases its genomic RNA and multiplies using the molecular structures of the cell. Transmembrane Serine Protease 2 (TMPRSS2) and lysosomal cathepsin activate SARS-CoV-2 entry [27].

### 1.3. Viral Proteases: Crucial Antivirals Therapeutic Targets

The main protease (Mpro) and papain-like protease (PLpro) are the most pursued viral proteins as SARS-CoV-2 antiviral drug targets [21]. They have been shown to be the most promising in inhibiting viral replication. The action of Mpro and PLpro is directed towards the proteolytic digestion of the viral polyproteins pp1a and pp1ab which leads to the formation of single viral proteins functional for the formation of the replication complex [28]. Specifically, three sites with the recognition sequence “LXGG ↓ XX” are cleaved by PLpro.

Shin et al. [29] reported an essential additional PLpro role concerning the dysregulatory action of the host’s immune response through an effective impairment of the antiviral effect of the host’s type I interferon. This intervention is mediated through its deubiquitinating and de-ISG15-ylating (interferon-induced gene 15) activities, respectively. Fu et al. [30] proved that SARS-CoV-2 PLpro intervenes to cleave ISG15 and induce modifications of polyubiquitin from cellular proteins. Therefore, inhibition of PLpro can lead to the accumulation of ISG15 conjugates and conjugates of polyubiquitin. This study reinforced the evidence from several reports suggesting substantial differences of PLpro in acting on substrates that concern SARS-CoV or SARS-CoV-2 replication. In fact, contrary to SARS-CoV PLpro, which prefers ubiquitinated substrates, SARS-CoV-2 PLpro prefers ISGlyated proteins as substrates [30]. The peculiar characteristic of PLpro is its belonging to a membrane-anchored multidomain protein named nonstructural protein 3 (nsp-3), which constitutes a fundamental component of the replicase−transcriptase complex.

As SARS-CoV-2 PLpro has been shown to have pleitropic roles, this makes it a promising target for antiviral drugs.

## 2. First Evidence from Deploying Therapeutics: SARS-CoV-2 Targets and Antivirals’ Mechanisms of Action

### 2.1. Post-Exposure Treatment Options: Lessons from ECDC Guidelines

Initial efforts aimed at developing drugs against COVID-19 have mainly focused on commonly adopted antiviral drugs. However, these strategies have had limited success [31].

The Infectious Diseases Society of America (IDSA) has developed and continuously updated an algorithm for the treatment of several forms of the disease: post-exposure prophylaxis, ambulatory care, moderate, severe, and critical care hospitalizations [32]. The major proposed treatments consist of remdesivir and favipiravir as inhibitors of viral RNA polymerases and, subsequently, of viral RNA production, corticosteroids such as dexamethasone, JAK-STAT pathway modulators, and other systemic therapeutic agents.

Hydroxychloroquine, an antimalarial drug, administered alone or in combination with azithromycin is not recommended against COVID-19. The rationale under this agent investigation comprises several anti-SARS-CoV-2 mechanisms of action, including the inhibition of the fusion of the viral particle with the host cell membrane. It is believed to inhibit the action of one of the most important cathepsins, cathepsin L, responsible for the cleavage of Spike proteins in endolysosomes, required for the viral entry into the host cells. Nonetheless, the effect of hydroxychloroquine was demonstrated to be less potent than expected, protease TMPRSS2 being the major determinant of spike proteins’ cleavage [33,34,35]. It was rapidly approved by FDA in March 2020 to treat hospitalized patients weighing at least 50 kg.

Unfortunately, after three months, the Emergency Use Administration (EUA) was dismissed and the clinical use revoked. The RECOVERY trial, designed to compare hydroxychloroquine to standard care for 4716 hospitalized patients with COVID-19 disease, failed to show any benefit in terms of 28 days mortality for hydroxychloroquine [36]. In fact, the Solidarity trial [37] and several metanalyses did not demonstrate positive effects in terms of morbidity or mortality with respect to standard management of hospitalized cases. Chi et al. [38] reported a network metanalysis of randomized controlled trials showing no differences in terms of mortality or mechanical ventilation use. Moreover, other studies found no differences regarding hospitalization risks in mild COVID-19 cases when comparing usual care or placebo with hydroxychloroquine use, instead, higher levels of side effects were noted [39,40,41,42].

Other non-antiviral possible agents recommended against COVID-19 include corticosteroids, interleukin-6 (IL-6) receptor antagonists, and monoclonal antibodies targeting Janus kinase-signal transducer and activator of transcription (JAK-STAT) pathway.

Tocilizumab and sarilumab are interleukin-6 (IL-6) inhibitors, considered for the treatment of patients responding to steroids in whom counterbalancing inflammatory signals such as proinflammatory cytokines seems to benefit. The therapeutic agents are under current FDA approval for several rheumatologic diseases. Tocilizumab, in particular, seemed to decrease clinical deterioration in terms of death, need for mechanical ventilation, ECMO, or intensive care unit (ICU) admission in several studies [43,44,45,46,47,48,49,50]. Notably, its use is currently suggested for COVID-19 hospitalized patients even though it seems indisputable that further research is needed. A randomized control trial performed to evaluate the outcome of 130 patients receiving either tocilizumab in intravenous administration or standard supportive care failed to demonstrate a clinical benefit in mortality after 28 days [45].

Tofacitinib is an oral inhibitor of Janus kinase 1 (JAK1) enzyme and of Janus kinase 3 (JAK 3), used in combination with methotrexate for the management of active rheumatoid arthritis [51]. Together with other agents such as baricitinib, it interferes with the JAK-STAT signaling pathway to reduce immunopathological reactions by restricting growth factor receptors stimulation [52]. Baricitinib administration has been associated with an increased risk of thromboembolism when compared with placebo in safety studies for rheumatological diseases and COVID-19 [51,53]. Moreover, it is generally known that COVID-19 might propagate coagulopathies with a thrombotic diathesis [54,55,56,57,58]. The use of tofacitinib is currently suggested for patients with severe COVID-19 on supplemental or high-flow oxygen [32].

Among ambulatory patients suffering from mild–moderate forms of COVID-19 and at high risk for progression to severe disease, the IDSA guidelines suggest casirivimab/imdevimab or sotrovimab, which represent monoclonal antibodies specifically directed against the spike protein [32]. 

Corticosteroid use has been largely discussed and biased by physicians both for outpatient management and for hospitalizations. Dexamethasone is a widely distributed agent and therapy duration, as concerning most immunosuppressive treatments, is a fundamental parameter. A large metanalysis comparing 21,350 patients from various clinical trials concluded that mortality is higher among patients treated with immunosuppressive steroid therapy and a time-span therapy duration of 3 to 12 days [59]. Notably, the longer the time under treatment, the higher the possibility of complications, such as prothrombotic ones. In general, the recommendation is to administer dexamethasone for 10 days and reserve longer treatment for selected and rare cases.

Evidence for corticosteroid application and efficacy in COVID-19 are generally represented by RCTs. Moderate level evidence has demonstrated overall mortality reduction in hospitalized patients being prescribed dexamethasone but other endpoints, such as ventilator-free days, a new need for invasive ventilation, and quality of life parameters were too statistically limited by low patient numbers to draw any meaningful conclusions [60,61,62,63,64,65]. In mild disease, case numbers were insufficient to derive conclusions. Dequin et al. [62] evaluated the effects of hydrocortisone administration on 21-day mortality in critically ill patients. The study was terminated with a low number of patients, like other similar studies (149 enrolled), and low dose hydrocortisone was demonstrated as non-superior to placebo with primary outcomes being mortality, mechanical ventilation use, and high-flow oxygen therapy. Secondary ones, comparable to the other RCTs, were the need for tracheal intubation, the incidence of pronation, extracorporeal membrane oxygenation, inhaled nitric oxide, Pao2:Fio2 ratio, and nosocomial superimposed infections [62].

### 2.2. FDA Approved and Authorized Antivirals: Indications and Pharmacodynamics

**Figure 1:**
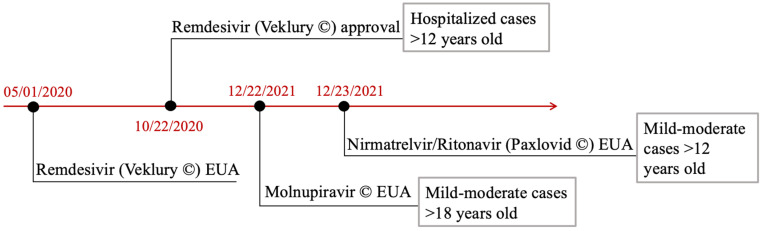
FDA timeline of antivirals approval and EUAs. Veklury^®^ EUA was formalized in January 2020. Its definitive approval occurred in October 2020. Molnupinavir and Paxlovid^®^ EUAs followed in December 2021. Indications are also presented, in gray-outlined boxes, on the side of each authorized therapeutic agent. Abbreviations. FDA: Food and Drug Administration; EUA: Emergency Use Authorization.

According to the clinical current evidence, the only food and drug administration (FDA)-approved antiviral for COVID-19 treatment is remdesivir. Molnupinavir and Paxlovid, a combination of nirmatrelvir and ritonavir are currently under Emergency Use Authorizations (EUAs). An events timeline is represented in Figure 1.

Favipiravir, with C_5_H_4_FN_3_O_2_ as formula and initially studied as anti-COVID-19 treatment by Chinese researchers [66], is a direct inhibitor of the viral RNA-dependent RNA-polymerase. It has never reached any authorization or approval by either FDA or European Medication Agency (EMA) due to the paucity of studies and inconclusive results [67,68,69,70]. In addition, the combination lopinavir/ritonavir has been tested in several trials but unfortunately, it has never been authorized for administration. The biochemical complete formula is C_37_H_48_N_6_O_5_S_2._ The combination is constituted by antiretrovirals used for the treatment of HIV infection. The molecular basis of action is protease inhibition. Several randomized controlled trials were conducted to evaluate hospitalized patients’ responses compared to standard care. Cao et al. [71] did not find any significant differences in terms of mortality at 28 days and of time to clinical improvement.

Similarly, the RECOVERY trial, a larger trial involving 176 hospitals from UK, did not find any differences in time until discharge alive from hospital or in the number of alive patients at discharge in 28 days [72]. Many other trials have reached the same outcomes; thus, the antiviral combination is not currently recommended for use in any of the clinical settings [73,74,75,76].

Remdesivir, structural formula C_27_H_35_N_6_O_8_P, (Veklury^®^) is an adenosine nucleotide prodrug, also called ProTide, of the 1′-cyano-substituted adenosine C-nucleoside (GS-441524) approved under Emergency Use Authorization by the Food and Drug Administration (FDA) in May 2020 and formally approved in October 2020.

Clinical indications include COVID-19 hospitalized adults and patients aged ≥12 years weighing ≥40 kg while the Emergency Use Authorization (EUA) also included hospitalized pediatric patients weighing 3.5 kg to <40 kg or aged <12 years and weighing ≥3.5 kg [77]. 

The antiviral mechanism of action is incorporation into viral RNA to inhibit coronaviruses synthesis. In particular, once entered the host cell, the prodrug GS-441524 is metabolized into GS-441524 triphosphate, the pharmacologically active metabolite GS-443902, i.e., a triphospate nucleotide, referred to by some authors as remdesivir triphosphate (RTP) [78,79]. It is then recognized by RNA-dependent RNA polymerase (RdRp) and incorporated into the growing substrate RNA. After that, the polymerase expands the RNA product by three more nucleotides before stopping. This mechanism, also referred to as stalling, is found to be specific to SARS-CoV-2 polymerase, more than other coronaviruses as Ebola viral polymerase stalling has been demonstrated after five RNA nucleotides [80,81,82]. Another favorable point is that, by provoking a stalling phenomenon, rather than a termination of the elongation, the virus avoids the exonucleolytic coronaviruses proofreading activity [83,84]. This mechanism has been previously demonstrated by in vitro studies and animal studies [85,86]. The recommended posology is constituted by an intravenous 5 mg/kg dose on the first day and 100 mg for 5 to 10 days. Especially in patients with oxygen saturation in ambient air falling under 94%, three randomized clinical trials were performed and failed to show a mortality benefit at 28 days but patients who were administered remdesivir underwent greater clinical improvements at 28 days than patients who underwent standard care [87,88,89].

Concerning critical patients on invasive ventilation or extracorporeal membrane oxygenation (ECMO), they neither received benefits in terms of mortality nor in recovery time. The same applies to patients with mild–moderate disease, i.e., patients with oxygen saturation >94% without supplemental oxygen [32].

On 23 December 2021, the FDA issued an Emergency Use Authorization (EUA) for Merck’s molnupiravir (C_13_H_19_N_3_O_7_) for the treatment of mild-to-moderate COVID-19 in non-hospitalized adults at high risk for progression to severe disease or without alternative options [90]. Molnupinavir is not indicated for patients younger than 18 years of age because it may affect bone and cartilage growth.

The posology recommendation was stated as four capsules, each consisting of 200 milligrams of drug, twice a day for five days. The mechanism of action is quite similar to that of remdesivir. It is intracellularly metabolized into an analog of cytidine, β-D-N^4^-Hydroxycytidine 5′-triphosphate (or NHC-TP). The RdRp enzyme incorporates it into its RNA leading to mutagenesis to all downstream copies and not to the stalling mechanism. This is also called error catastrophe [91].

On December 22, 2021, the FDA released an EUA for Pfizer’s Paxlovid (nirmatrelvir tablets and ritonavir tablets, co-packaged for oral use) for the treatment of mild-to-moderate COVID-19 infection in non hospitalized adults and pediatric patients (>12 years of age) who were at high risk for disease progression [92]. Paxlovid is administered as three tablets taken together orally every 12 h for a total of five days. 

Nirmatrelvir (PF-07321332) is an inhibitor of 3C-like protease (3CL^PRO^), with a structural formula of C_23_H_32_F_3_N_5_O_4_, and is a viral enzyme responsible for the cleavage of polyproteins 1a and 1ab of SARS-CoV-2, containing the protease itself and 15 other non-structural proteins [93,94]. By inhibiting their cleavage, the virus cannot be reproduced as the viral replication is stopped. Ritonavir, being a potent P450 (CYP) 3A4 inhibitor, is important for maintaining higher concentrations of nirmaltrevir by reducing its metabolism. Similar pharmacokinetic mechanisms can be observed for HIV medications.

**Figure 2:**
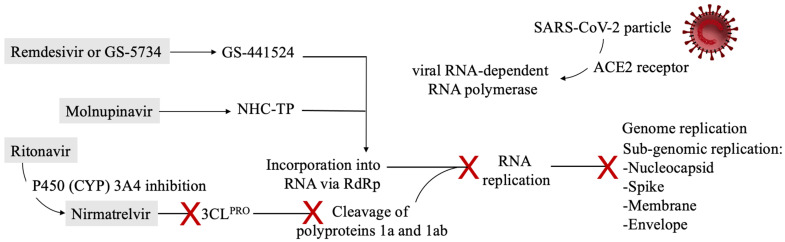
Antivirals’ intracellular metabolism and mechanisms of action are depicted in the figure. SARS-CoV-2 particles, after entering into the host cell, can reproduce via RNA-dependent RNA polymerase (RdRp). The enzyme is normally responsible for viral RNA replication, but it incorporates the active nucleotides analogs remdesivir and molnupinavir, either stalling or terminating the replication because of mutagenesis. Ritonavir is administered with nirmatrelvir to slow down its metabolic inactivation. The antiviral nirmatrelvir inhibits viral proteases responsible for the cleavage of structural proteins. Antivirals are depicted in graay boxes. Abbreviations. SARS-CoV-2: severe acute respiratory syndrome coronavirus 2, NHC-TP: β-D-N^4^-Hydroxycytidine 5′-triphosphate, CYP: cytochrome P450, RNA: ribonucleic acid, ACE2: angiotensin-converting enzyme, 3CLPRO: 3C-like protease.

Antivirals’ mechanisms of action are represented in Figure 2.

## 3. Potential Impact of Antivirals on the Cardiovascular System and Pre-Existing Cardiovascular Conditions

### 3.1. Remdesivir Cardiac Pro-Arrhythmogenic and Hypotensive Effects

Among the most frequent side effects of remdesivir seem to appear anemia, gastrointestinal symptoms, hypertransaminasemia, cutaneous rashes, and kidney injury, other than constitutional local reactions such as infusion-related ones [95]. Notably, patients may also experience hypotension and cardiac conduction disturbances, as documented by electrocardiographic monitoring. One of the hypothesized explanations is attributed to the structural similarity to adenosine and to the binding of the drug to multiple adenosine receptors.

Not every adenosine analog is capable of interacting with adenosine receptors. Nonetheless, pharmacological studies were performed to assess the potential binding of remdesivir and its metabolites to adenosine A1, A2, and A3 receptors. GS-441524 metabolite has been predicted to bind to adenosine receptors A1, A2, and A3 and to adenosine kinase while GS-443902, i.e., remdesivir triphosphate, was demonstrated to not interact with them. Adenosine is a potent vasodilator and both a pro-arrhythmogenic and anti-arrhythmogenic agent, depending on the structural heart disease, thus there has been a tendency to focus on its analogs’ potential adverse effects [96].

Remdesivir can exert an impact on cardiac tissue by inducing electrocardiographic changes such as bradycardia, T-wave abnormalities, atrial fibrillation, and prolonged QT interval [95]. In addition, a few cases of cardiac arrest were demonstrated following remdesivir infusion.

Two main mechanisms are responsible for these changes: the compensatory catecholamines release after adenosine-induced hypotension, which predisposes to ventricular tachycardia and ventricular fibrillation, and the induction of inhomogeneity in ventricular refractoriness as determined by a case report in which a patient suffering from stable ventricular tachycardia was administered adenosine and subsequently experienced ventricular fibrillation [97]. There also are reports of idiosyncratic reactions to adenosine in the absence of structural heart diseases with induction of ventricular fibrillation [98].

Chow et al. [99] reported the case of a pediatric patient administered with a single therapeutic dose of remdesivir because of severe acute COVID-19. Over the following hours, the patient experienced persistent sinus bradycardia, whereby the following administrations were suspended.

Another possible molecular basis of this explanation is the damage to mitochondrial RNA polymerase. A preclinical study using in vitro human pluripotent stem cell-derived cardiomyocytes employed as substitutes of human cardiomyocytes revealed this electrophysiologic toxicity [100]. Multielectrode array (MEA) method was used to assess the automaticity and electrocardiographic parameters, such as QT interval prolongation on cardiomyocytes. Chloroquine was used as a positive control as it induces cardiotoxicity. Results from this study corroborate the toxicity hypothesis: dose-dependent prolongations of field potential duration (FPD), reduced Na^+^ peak amplitudes, and spontaneous beating rates, suggesting a pro-arrhythmogenicity in human cardiomyocytes [100]. At higher doses, remdesivir was in fact demonstrated to cause QT prolongation.

Authors from this study advise close monitoring of cardiac conduction function, in particular of the QT interval, in all patients, especially the predisposed ones, under remdesivir treatment for COVID-19.

From a clinical point of view, several other cases of cardiovascular side effects were registered. Two randomized controlled trials, Mulangu et al. [101] and Wang et al. [102], despite differences in statistical power and bias addressing, each reported a case of severe toxicity after remdesivir administration. In the former study, 681 patients suffering from Ebola viral disease in the Democratic Republic of Congo were enrolled and divided into four groups of treatment. Of 681 patients, 175 received remdesivir and in one patient, cardiac arrest was reported [101].

The latter, a randomized, double-blind, placebo-controlled, multicenter trial involving ten hospitals in Hubei, China compared 237 patients, of which 158 were assigned to remdesivir treatment and 79 to placebo. Adverse events were reported in 102 (66%) of 155 remdesivir recipients and in one of them, hypotension and cardiac arrest was noted [102].

VigiBase is an individual case safety report database of the World Health Organization (WHO) evaluated by a group of scientists from Seoul, Korea [103]. Cardiac arrest (adjusted odds ratio (aOR): 1.88, 95% confidence interval (CI): 1.08–3.29), bradycardia (aOR: 2.09, 95% CI: 1.24–3.53), and hypotension (aOR: 1.67, 95% CI: 1.03–2.73) were associated with remdesivir. In particular, from a total number of 2107 individual case safety reports, 93 out of 2107 patients (i.e., 4.41%) experienced cardiac arrest, 79 (3.75%) experienced bradycardia, 19 patients suffered from cardiogenic shock, 48 patients reported hypotension and about a hundred patients experienced electrophysiological conduction changes, including atrial fibrillation, ventricular tachycardia, and fibrillation, sinus tachycardia, QT prolongation, atrial flutter, torsade de points, and others [103].

### 3.2. Electrocardiographic Changes after Ritonavir Administration

The administration of lopinavir/ritonavir also revealed adverse drug reactions such as ventricular tachycardia, ventricular fibrillation, torsade de points, long Q-T syndromes, and cardiac arrest [104].

Concerning ritonavir administration, it is a well known pro-arrhythmogenic when administered alone or in combination with saquinavir (another anti-HIV medication). In particular, the FDA warned physicians about alterations of QT intervals leading to torsades de points and prolonged PR intervals, leading to heart blocks [105].

Ritonavir-linked cases of bradycardia were demonstrated after combination therapy administration of lopinavir/ritonavir. A French observational prospective study [106] reported 9 cases of bradycardia (22%) among 41 COVID-19 positive patients, admitted to the local hospital intensive care unit and administered lopinavir/ritonavir combination drug for at least 48 h. Of these 9 cases, 8 had sinus bradycardia and 1 had a third-degree atrioventricular block. All the cases were resolved after therapy discontinuation or antiviral dose-reduction [106]. The authors also explained the possibility of inflammatory damage from COVID-19 associated with higher intestinal absorption of the antiviral combination and thus of the possible side effect. Several other studies also confirmed the association of lopinavir/ritonavir administration for COVID-19 and new arrhythmic events, including atrial fibrillation, atrial flutter episodes, and long-QT syndromes [107,108].

### 3.3. Drug–Drug Interactions between Antivirals and Cardioactive Medications

In addition, concerns about concomitant administration of cardiovascular medications with ritonavir in combination with nirmaltrevir arose [109]. Ritonavir-boosted nirmatrelvir (Paxlovid) has significant and complex drug–drug interaction potential, primarily due to the ritonavir component of the combination. 

Ritonavir is generally known to induce cytochromes CYP2B6, CYP2C19, CYP2C9, and CYP1A2, and, more relevantly, to strongly inhibit CYP3A4 and CYP2D6, this leading to severe drug–drug interactions. Cardioactive medications, especially certain antiplatelets, statins, anticoagulants, and anti-arrhythmic agents are cytochromes substrates and, in several studies, were demonstrated to be partially modified by ritonavir in their pharmacokinetics [109].

Regarding antiplatelets, there is a tendency to replace prasugrel with clopidogrel, as the former requires metabolic activation by CYP3A4 and CYP2B6. The concomitant administration with ritonavir seems in fact to decrease several pharmacokinetic parameters including AUC_0–6h_ and maximum concentration (C_max_) of prasugrel active metabolites [110,111,112]. Aspirin, cangrelor, and other antiplatelet agents can be instead safely co-administered with ritonavir.

Lipid-lowering drugs also have been demonstrated to interact with ritonavir administration. Statins in particular are metabolic substrates for hepatic cytochrome CYP450. More specifically, lovastatin, simvastatin, and atorvastatin i.e., lipophilic statins, are metabolized by CYP3A4.

One of the examined studies presented a direct correlation between the administration of ritonavir-boosted saquinavir and changes in simvastatin pharmacokinetics. Of note, its AUC and C_max_ were shown to be increased by 30-fold [113].

Ezetimibe, hydrophilic statins, and fibrates can be instead administered without particular precautions. The only suggestion is to closely monitor possible side effects [109].

Non-vitamin K oral anticoagulants were also studied for the strong interactions. Rivaroxaban and apixaban are metabolized through a variety of pathways, including CYP3A4 and BCRP. Rivaroxaban is a direct factor Xa inhibitor and a life-saving drug, indicated for atherothrombotic event prevention after an acute coronary syndrome or in patients suffering from coronary artery disease (CAD) or peripheral artery disease (PAD) at high ischemic risk. Pharmacological in-vivo studies have demonstrated that concomitant administration with ritonavir strongly increases rivaroxaban AUC and C_max_. In a study by Mueck et al. [114], a 153% increase (95% CI: 134% to 174%) of rivaroxaban AUC in concomitant administration with ritonavir was noted. Edoxaban, unfractionated heparin, enoxaparin, and fondaparinux do not exhibit significant interactions with ritonavir.

Thus, particular attention on possible alterations of coagulative profile should be paid for patients in anticoagulation therapy and ritonavir.

In healthy subjects, 10 days of lopinavir/ritonavir therapy increased CYP2C9 activity by 29% and CYP1A2 by 43% [115]. The induction of these cytochromes by L/R may result in increased warfarin metabolism and a reduction in the international normalized ratio (INR). 

Ritonavir coadministration is also contraindicated with dronedarone, encainide, flecainide, propafenone, and quinidine [116].

No significant interactions were instead generally evaluated for beta-blockers, vasodilators diuretics, ACE inhibitors, and angiotensin II receptor blockers (ARBs).

Concerning the metabolism of Nirmatrelvir, a general consideration to convey is the paucity of data and clinical or pharmacological studies about antiviral interactions and side effects. It is claimed that the agent’s metabolism can be decreased when combined with certain calcium channel blockers (CCBs) such as verapamil, nilvadipine, and Nicardipine, with lovastatin and amiodarone [117,118].

The most important authorized and approved antivirals interactions and side effects are tabulated below, in Table 1.

## 4. Two Pivotal Questions to Find a Balance between Cardiovascular Implications and Antivirals’ Safety Profiles

### 4.1. Is There Any Chance the Antivirals’ Side Effects Reports Are Biased by COVID-19 Cardiovascular Direct Complications?

Typically, patients with severe symptomatic COVID-19 and predisposing cardiovascular conditions present worse clinical pictures. In particular, patients with coronary artery disease (CAD) or myocardial failure are more prone to develop major cardiac injury, a risk factor for myocardial impairment that requires hospitalization and intensive care unit admission. The molecular basis for it is dependent upon the abundance of ACE receptor surface cell expression in cardiovascular diseases, a gateway for the virus to enter the host cell [119]. Virus anchoring to the cell surface may induce alterations in the physiological pattern of the renin-angiotensin-aldosterone system (RAAS), an enzymatic cascade involved in blood pressure control. This biochemical homeostasis is reached by regulating body fluid and systemic vascular resistance. ACE1 (angiotensin I-converting enzyme) forms angiotensin II (Ang-II) from angiotensin I (Ang-I). Ang-II binds and activates Angiotensin Type 1 Receptor (AT1R), which leads to vasoconstriction, inflammation, and fibrosis [120]. ACE2 converts Ang-II into angiotensin 1–7 (Ang 1–7), which has vasodilating and anti-inflammatory action by anchoring to the MAS receptor (MAS-R). ACE2 also cleaves Ang-I into angiotensin-1–9, which is converted into Ang 1–7 by ACE. ACE2 plays the main role in the regulation of abnormal activation of RAAS [121]. In fact, an increase in ACE2/ACE1 ratio had a primary prophylaxis role in preventing endothelial dysfunctions and vascular constriction. Elevated values of ACE2 may indicate that patients are more prone to achieving SARS-CoV-2 infection and is a negative prognostic element in SARS-CoV-2 related cardiovascular diseases [122,123].

Aside from COVID-19 thrombotic diathesis, disease pro-arrhythmogenicity has also been studied in detail. A worldwide analysis of novel arrhythmia cases during COVID-19 infection has drawn attention to the electrophysiologic complications, including cardiac arrest and polymorphic ventricular tachycardia [124]. A retrospective, 29 institution-comprehensive study, reported that, among 4526 patients hospitalized for COVID-19, 827 developed cardiac arrhythmias during hospitalization of which the majority presented with pre-existing medical conditions such as atrial fibrillation, ventricular tachycardia, and heart failure [124]. Ventricular arrhythmias were associated with significantly higher mortality rates.

Therefore, it is quite evident that distinguishing the clinical syndrome caused by COVID-19 and the side effect caused by the antiviral is not so linear. In particular, one point in favor of the administration of Paxlovid and Molnupinavir is the authorization for short therapeutic cycles of 5 days only.

An important clarification should also be made with respect to several studies reporting clinical outcomes from coadministration of antivirals with hydroxychloroquine: often results may be misinterpreted because of the tendency of hydroxychloroquine to not exert any clinical benefit, as largely demonstrated, but to cause several side effects.

### 4.2. Do These Complications Matter? Monitoring, Clinical Suggestions, and Continuous Updating May Be the Answer

Cardiovascular complications are distributed in various forms of COVID-19 and various populations [125,126,127,128]. The tendency is to be cautious with repurposed drugs administration, for COVID-19. However, given the lack of available data, concerns have been raised within the scientific community especially as the mechanism of action of some antivirals remains unclear [58].

Our general recommendations for outpatients and hospital management of antivirals administration, from a cardiovascular point of view, could be summarized in the following bullet points:

- In all patients and especially the predisposed ones, continuous cardiac rhythm monitoring, especially checking the QT interval length should be provided;
- Remdesivir should be withdrawn in presence of known congenital long QT syndrome or baseline QT prolongation;
- Other QT-prolonging agents should be avoided, whenever possible, during remdesivir administration;
- Drug monitoring and critical switching to cardioactive alternatives should be made before ritonavir administration;
- INR monitoring in patients on vitamin K oral anticoagulants and ritonavir coadministration should be frequently executed;
- Where possible, prasugrel administration should be substituted by clopidogrel and hydrophilic statins should be preferred during ritonavir therapy course.

It is evident and crucial that more studies are needed to confirm antivirals’ safety for the cardiovascular system. Nonetheless, within the narrow therapeutic window proposed by the FDA, they should be well-tolerated, also given the rejection of approval for pediatric patients as another sign of caution in the authorizations’ communication.

Our suggestion is to strictly follow the FDA indications, respect the correct weight-adjusted posology and contraindications, and to continuously update the knowledge and awareness of novel discovered interactions and reported side effects.

## 5. Conclusions

The pandemic has given rise to a good response from the scientific community with the availability of multiple therapeutic agents and vaccines. Vaccines continue to provide sufficient protection from spread and contagion although their response varies with disease variants [58,129,130]. There is an ongoing push towards biological agents and retrovirals to reduce the severity and duration of symptoms with mixed results. As with many advances in therapeutics, ongoing monitoring, reporting, and updates are required to ensure safety and efficacy for the generalized use of these medications especially in patients with multiple comorbidities.

## Figures and Tables

**Table 1:** Therapeutic agents’ profiles of potential cardiovascular toxicities and drug–drug interactions with cardioactive medications. In the last column, metabolic properties of the agent, responsible for interactions, are summarized. Abbreviations. DDIs: drug–drug interactions, CYP: cytochrome P450, VT: ventricular tachycardia, VF: ventricular fibrillation, LQTs: long Q-T syndromes.

Antiviral Agent	Cardiac Side Effects	Relevant DDIs with Cardioactive Medications	Metabolic Properties
remdesivir (Veklury^®^)	Pro-arrhytmogenic: bradycardia, T-wave abnormalities, atrial fibrillation, prolonged QT interval, VT, VF, cardiac arrest. Hypotension.	Not relevant.	Minor substrate of cytochrome CYP3A4.
molnupinavir^®^	Not relevant; mainly gastrointestinal ones.	No substantial risk, lack of clinical interaction studies.	/
nirmatrelvir (Paxlovid^®^)	Not relevant, paucity of data.	Paucity of data; possible DDIs with amiodarone, verapamil, nilvadipine, nicardipine, lovastatin.	Minor substrate of CYP3A4.
ritonavir (Paxlovid^®^)	Pro-arrhythmogenic: LQTs, torsades de points, bradycardia, VT, VF, atrial fibrillation, atrial flutter, cardiac arrest.	Ticagrelor, simvastatin, rivaroxaban, lercanidipine, anti-arrhythmics (dronedarone, encainidie, flecainide, propafenone, quinidine), ivabradine.	Inducer of CYP2B6, CYP2C19, CYP2C9, and CYP1A2, strong inhibitor of P450 3A4 and CYP2D6.

## Data Availability

Not applicable.

## Abbreviations

ACE1	angiotensin I-converting enzyme,
ACE2	angiotensin-converting enzyme 2,
Ang-II	angiotensin II,
Ang-I	angiotensin I,
Ang 1–7	angiotensin 1–7,
APS	antiphospholipid syndrome,
ARBs	angiotensin II receptor blockers,
AT1R	angiotensin type 1 receptor,
AUC_0–6h_	area under the curve,
CAD	coronary artery disease,
CCBs	calcium channel blockers,
Cmax	maximum concentration,
CSG	Coronaviridae Study Group,
CYP	cytochrome P450,
DDIs	drug–drug interactions,
E	envelope,
ECMO	extracorporeal membrane oxygenation,
EMA	European Medicines Agency,
EUA	Emergency Use Administration,
FDA	Food and Drug Administration,
JAK-STAT	Janus kinase and signal transducer and activator of transcription,
Kb	kilobases
ICU	intensive care unit,
IDSA	Infectious Diseases Society of America,
IL-6	interleukin-6,
INR	international normalized ratio,
LQTs	long Q-T syndromes,
M	membrane,
MERS-CoV	Middle East respiratory syndrome coronavirus,
Mpro	main protease,
N	nucleoprotein,
NHC-TP	β-D-N4-hydroxycytidine 5′-triphosphate,
nsp-3	nonstructural protein 3,
ORF	open reading frames,
PAD	peripheral artery disease,
PLpro	papain-like protease,
RAAS	renin-angiotensin-aldosterone system,
RCTs	randomized clinical trials,
RdRp	RNA-dependent RNA polymerase,
RNA	ribonucleic acid,
RTP	remdesivir triphosphate,
SARS-CoV	severe acute respiratory syndrome coronavirus,
SARS-CoV-2	severe acute respiratory syndrome coronavirus 2,
TMPRSS2	transmembrane serine protease 2,
UTR	untranslated regions,
VF	ventricular fibrillation,
VT	ventricular tachycardia,
WHO	World Health Organization,
WT	wild-type,
3CLPRO	3C-like protease.
